# Bilateral Lung Transplantation for Patients With Destroyed Lung and Asymmetric Chest Deformity

**DOI:** 10.3389/fsurg.2021.680207

**Published:** 2021-08-10

**Authors:** Bingqing Yue, Shugao Ye, Feng Liu, Jian Huang, Yong Ji, Dong Liu, Jingyu Chen

**Affiliations:** Wuxi Lung Transplant Center, Department of Thoracic Surgery, Wuxi People's Hospital Affiliated to Nanjing Medical University, Wuxi, China

**Keywords:** destroyed lungs, asymmetric chest deformity, bilateral lung transplantation, extracorporeal membrane oxygenation, lung function

## Abstract

**Background:** Destroyed lung can cause mediastinal displacement and asymmetric chest deformity. Reports on bilateral lung transplantation (LT) to treat destroyed lung and asymmetric chest deformity are rare. This study presents our surgical experience of bilateral LT among patients with destroyed lung and asymmetric chest deformity.

**Methods:** Six patients with destroyed lung and asymmetric chest deformity who underwent bilateral LT at our center from 2005 to 2020 were included in the study. Demographic data, technical data, perioperative details, and short-term follow-up data were reviewed.

**Results:** Three patients underwent bilateral LT via anterolateral incisions in the lateral position without sternal transection, while three patients underwent bilateral LT via clam-shell incisions in the supine position with sternal transection. Only one patient required intraoperative extracorporeal membrane oxygenation. Four patients underwent size-reduced LT. In the other two patients, we restored the mediastinum by releasing mediastinal adhesions to ensure maximal preservation of the donor lung function. Patients in the lateral position group had a higher volume of blood loss, longer operation time, and longer postoperative in-hospital stay than those in the supine position group. However, these differences were not statistically significant. Postoperative computed tomography in the supine position group revealed that the donor lungs were well expanded and the mediastina were in their original positions.

**Conclusions:** Although bilateral LT in patients with destroyed lung and asymmetric chest deformity is high risk, with sufficient preoperative preparation and evaluation, it is safe and feasible to perform bilateral LT for selected patients. For patients without severe chest adhesions, releasing the mediastinal adhesions and restoring the mediastinum through a clam-shell incision in the supine position is a simple and effective method to maximally preserve the donor lung function without pneumonectomy or lobectomy.

## Introduction

Destroyed lung refers to the clinical destruction of a lung lobe or an entire lung. It may manifest as extensive cavities, fibrosis, and bronchial stenosis or dilation, and the affected lung is susceptible to recurrent suppurative or fungal infections (1, 2). It can cause mediastinal displacement and asymmetric chest deformity, resulting in high-risk surgery. Moreover, patients with destroyed lung have short life expectancies and poor prognoses, and its treatment poses a significant clinical challenge. Lung volume reduction surgery is a procedure performed to manage patients with lesions limited to a single lung (3). In patients with end-stage destroyed lung, both lungs suffer severe deterioration in function, and lung transplantation (LT) is often considered the only effective treatment. To date, there have been no reports on bilateral LT for patients with destroyed lung and asymmetric chest deformity without pneumonectomy or lobectomy. Herein, we report on six patients with asymmetric chest cavities due to destroyed lung who underwent bilateral LT and present our surgical and clinical experience.

## Patients and Methods

### Study Design and Data Collection

We retrospectively analyzed the medical records of all consecutive patients with destroyed lung and asymmetric chest deformities who underwent LT at the Lung Transplantation Center of Wuxi People's Hospital from 2005 to 2020. Information collected from all six patients included demographic data, surgical details, and short-term follow-up data.

The study was conducted in accordance with the Declaration of Helsinki (2000), and its later amendments, and the Declaration of Istanbul (2008). All patients or their next of kin provided written informed consent. The study protocol was approved by the Institutional Ethics Committee of Wuxi People's Hospital. The surgical operations were performed by two experienced senior thoracic surgeons (Chen and Ye).

### Perioperative Assessment

Preoperative investigations included blood examination, assessment of infection status, lung function evaluation, functional assessment of other vital organs, and anesthetic evaluation. The surgical indications for patients with destroyed lung and asymmetric chest deformities were mainly based on the 2006 International Society for Heart and Lung Transplantation guidelines (4). All patients underwent multidisciplinary preoperative discussion with healthcare professionals, including thoracic surgeons, respiratory physicians, cardiologists, anesthesiologists, intensive care unit (ICU) doctors, physical therapists, nutritionists, and ethics committee members.

The ABO blood groups of the donors and recipients were identical preoperatively. Preoperative chest X-ray or computed tomography (CT) examinations did not reveal any pulmonary infections or other pulmonary diseases among the donors, and their oxygenation indexes were >300.

### Surgical Techniques

One patient who underwent surgery in the lateral position had high pulmonary arterial pressure, and venoarterial extracorporeal membrane oxygenation (V-A ECMO) was performed intraoperatively via the right femoral artery and vein catheterization. The other five patients underwent uneventful surgeries without the need for ECMO. The coagulation time was controlled within 150–200 s.

The surgeries were performed with the patients in the lateral position via an incision made in the anterolateral fifth intercostal space without sternal transection. First, the destroyed lung was removed and replaced with the donor lung. The bronchus and pulmonary artery and vein were then sequentially anastomosed. After the donor lung was well ventilated and satisfactorily oxygenated, the chest remained temporarily open. Subsequently, the patient was turned over, and the contralateral LT was performed. The second donor lung was implanted, and the chest was closed directly after transplantation. Next, the patient was again turned over to reduce the volume of the transplanted lung on the side of the destroyed lung according to the size of the patient's chest cavity. Finally, a chest drainage tube was inserted, and the chest was closed at the end of the operation.

**Figure 1:**
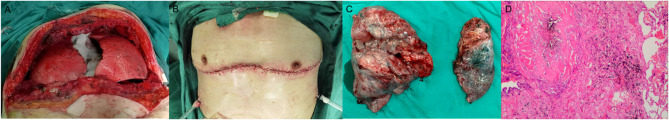
Surgical and pathological images of patient 1. **(A)** The mediastinum was restored after the release of mediastinal adhesions, and the sizes of the bilaterally transplanted lungs were moderate, which were consistent with the size of the recipient's chest. **(B)** The operation was performed in the supine position with a clam-shell incision along the 4th intercostal space. **(C)** A surgical specimen of the recipient's lungs. Note the significant compensatory enlargement of the recipient's right lung (left) and the damaged left lung (right). **(D)** Postoperative pathological examination showed silicosis nodules and silica dust particle deposition.

The surgical method involving sternal transection in the supine position was performed in the fourth anterior intercostal space through a clam-shell incision. Then, the destroyed lung was first dissociated and removed, and the heart was moved towards the midline to match the patient's chest cavity and the donor lung's size. We tried to avoid reducing the donor lung's volume during implantation. After the donor lung was well ventilated and satisfactorily oxygenated, the contralateral emphysematous lung was removed, and the other donor lung was implanted. Next, a chest drainage tube was inserted, and the chest was closed at the end of the operation (Figure 1).

### Postoperative Management

Postoperatively, all patients were transferred to the thoracic ICU. Immunosuppressive drugs administered included mycophenolate mofetil, tacrolimus, and prednisone. All patients received prophylactic antimicrobial and antiviral medications to prevent bacterial, fungal, and viral infections. Postoperative blood examinations, chest radiography, and bronchoscopy were performed regularly.

### Statistical Analysis

Continuous data with normal distributions are presented as means with their standard deviations. Categorical variables were evaluated using Fisher's exact test and descriptively analyzed using *t*-tests. All data were analyzed using SPSS version 16.0 (SPSS Inc., Chicago, IL, USA). Analysis items with *p* < 0.05 were considered statistically significant.

## Results

Six patients underwent bilateral LT during the study period; all were male and had a mean age of 39.3 ± 16.6 years. Two patients had destroyed lung as a result of tuberculosis. Preoperative examination showed no relapses of tuberculosis. The primary diagnoses of the other patients were silicosis, bronchiolitis obliterans syndrome (BOS), and bronchiectasis. One patient had a preoperative *Candida albicans* infection. Three patients had a destroyed left lung, and three had a destroyed right lung. In addition, four patients had mild-to-severe pulmonary hypertension. Case 4 had experienced a recurrent cough, sputum production, and wheezing for more than 20 years. Two years before admission, he was admitted to the hospital for emergency treatment multiple times due to dyspnea and was treated via tracheotomy and ventilator-assisted ventilation. Case 6 experienced respiratory failure due to a severe preoperative lung infection and was managed via tracheal intubation and mechanical ventilation (Table 1).

**Table 1:** General characteristics of patients with destroyed lungs.

**Case**	**Sex**	**Age (years)**	**The cause of destroyed lung**	**Fungal infection**	**Preoperative respiratory support**	**The side of destroyed lung**	**Pulmonary hypertension**	**Operation time**	**Operation type**	**Incision**	**Surgical position**
1	M	48	Silicosis	N	Nasal catheters	Left	N	2020.08	Bilateral LT	Clam-shell incision	Supine position
2	M	20	BOS	Y	Nasal catheters	Left	Mild	2019.12	Bilateral LT	Clam-shell incision	supine position
3	M	31	Bronchiectasis	N	Nasal catheters	Right	Severe	2016.09	Bilateral LT	Clam-shell incision	supine position
4	M	61	Tuberculosis	N	Invasive ventilator	Right	N	2015.10	Bilateral LT	Anterolateral incision	Lateral position
5	M	24	Tuberculosis	N	Non-invasive ventilator	Right	Moderate	2006.05	Bilateral LT	Anterolateral incision	Lateral position
6	M	52	Bronchiectasis	N	Invasive ventilator	Left	Mild	2005.03	Bilateral LT	Anterolateral incision	Lateral position

*BOS, bronchiolitis obliterans syndrome; LT, lung transplantation*.

Three patients underwent surgery in the lateral position via an anterolateral incision without sternal transection, and three patients underwent surgery via a clam-shell incision in the supine position with sternal transection. Intraoperatively, we found that the destroyed lung was severely consolidated and damaged, with extensive adhesions to the chest wall, mediastinum, and diaphragm, and compensatory emphysema on the contralateral side, with mediastinal displacement. Three patients in the lateral position group underwent size-reduced LT, with the right upper lobe, right lower lobe, and left upper lobe removed in each case, respectively. Only one patient in the supine position group underwent size-reduced LT, with the right middle lobe removed. The other two patients in the supine position group underwent intraoperative resection of mediastinal adhesions, and size reduction of the donor lungs was not required. The blood loss volume and operation time of the patients in the lateral position group were higher than those in the supine position group, but no statistically significant difference was observed (*p* > 0.05) (Table 2).

**Table 2:** Comparisons of intraoperative and postoperative characteristics between supine position group and lateral position group.

	**Supine position group**	**Lateral position group**
**Intra-operative data**		
Bleeding volume(mL)	1,800 ± 565.7	3,850 ± 1,626.3
Time (min)	400 ± 120.2	503.3 ± 106.0
Intraoperative EMCO	0	1
Size-reduced LT	1	3
**Post-operative outcomes**		
Postoperative bleeding	0	1
Postoperative tracheotomy	1	0
Hospital stay (days)	26.3 ± 3.5	36.3 ± 11.0
Survival	2	2

*EMCO, extracorporeal membrane oxygenation; LT, lung transplantation*.

**Figure 2:**
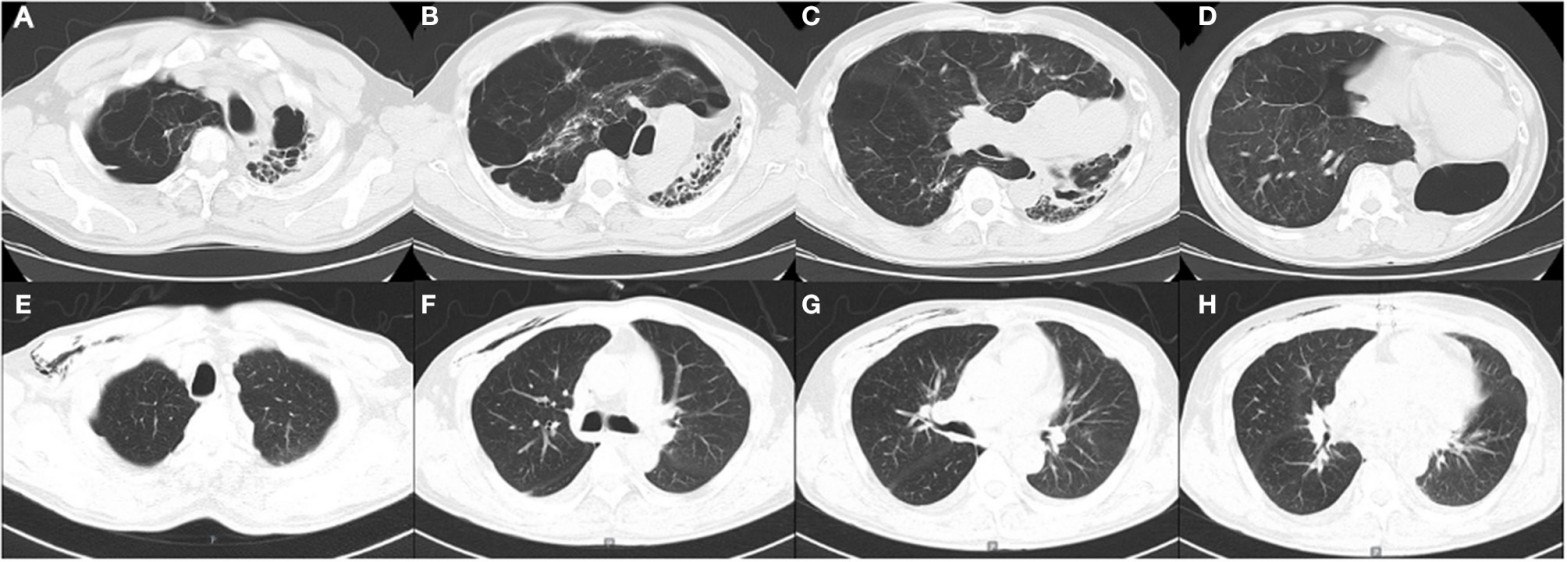
**(A–D)**. Preoperative computed tomography (CT) images of patient 1. **(E–H)**. Postoperative CT images of patient 1 one month after the surgery.

The mean duration of in-hospital stay in the lateral position group was higher than that in the supine position group but without a statistically significant difference (*p* > 0.05) (Table 2). Postoperatively, two patients with the history of tuberculosis received regular anti-tuberculous treatment, and their repeated sputum smear tests yielded negative results. One month postoperatively, a CT scan revealed that the donor lung was well expanded, and the mediastinum was reset in case 1 (Figure 2). One patient died in each group during the perioperative period. Case 3 had frequent preoperative infections and a history of vocal cord paralysis; although a tumor was ruled out, this severely affected his postoperative cough. Postoperatively, the patient found it difficult to expel sputum from the lungs via coughing, causing severe pulmonary infection, and a tracheotomy was performed because of decreased oxygenation. Finally, the patient died suddenly of severe hemoptysis. Case 4 was admitted to our hospital for rescue LT. Due to the severity of his preoperative condition, his respiratory muscles had drastically weakened. Postoperatively, sputum in the lungs could not be expelled via coughing, and bronchoscopy revealed a considerable amount of yellow pus in the bronchus. A sputum culture yielded *Acinetobacter baumannii, Pseudomonas aeruginosa*, and *Klebsiella pneumoniae*, and the patient died of septic shock and multiple organ failure 1 month postoperatively. The remaining patients were discharged.

## Discussion

Quality of life is severely reduced among patients with end-stage destroyed lung (5). LT may be the only effective way to save these patients' lives and improve their quality of life. The present study emphasizes the feasibility and practicability of two types of bilateral LT for patients with destroyed lung and asymmetric chest deformity. By removing the mediastinal adhesions and restoring the mediastinum, pneumonectomy or lobectomy were not required, thus enabling maximal preservation of donor lung function.

Various diseases (such as tuberculosis and bronchiectasis) can cause destroyed lung (6); recurrent tuberculosis and erratic tuberculosis treatments irreversibly damage the lung parenchyma (7). When affected by chronic tuberculosis and chronic inflammatory exudation, the destroyed lung and its surroundings may develop dense corpus callosum-like adhesions, which are challenging to separate and significantly increase the difficulty of surgery and the risk of bleeding. Moreover, immunosuppressive therapy after lung transplantation may lead to the recurrence of tuberculosis (8). In this study, we performed bilateral LT for two patients with a lung destroyed by tuberculosis. One patient died of septic shock and multiple organ failure 1 month postoperatively. This may have been due to the preoperative severity of his condition, which resulted in his respiratory muscles becoming weakened, preventing him from expelling sputum from his lungs via coughing; this led to a severe postoperative infection. The other patient was discharged without complication and had no recurrence of a lung destroyed by tuberculosis. We suggest that adequate preoperative blood preparation and surgical evaluation are required for patients with a lung destroyed by tuberculosis undergoing LT.

Bronchiectasis is another leading cause of destroyed lung, is a significant indication for LT, and accounts for approximately 30% of all LTs worldwide (9). Due to the higher incidence of preoperative respiratory failure and secondary pulmonary hypertension in patients with bronchiectasis, the mortality rate of patients with this condition on the transplant waiting list is higher than that of those with other diseases (10), and LT should be performed promptly for these patients. Chronic infections caused by bronchiectasis may result in multiple drug-resistant pathogens, making postoperative treatment difficult (11); bilateral LT is recommended to reduce the risk of postoperative autologous lung infections (12). Bronchiectasis causes bilateral lung damage and asymmetric chest cavity, which further increases the difficulty of surgery and postoperative treatment. In our study, cases 3 and 6 had severe preoperative lung infections. Case 3 had a history of vocal cord paralysis; thus, he could not expel sputum from the lungs via coughing, causing severe pulmonary infection; this may have been the main cause of his death. Case 6 cooperated very well with the postoperative treatment and recovered uneventfully. His quality of life has improved significantly, and he has survived for more than 6 years.

BOS after hematopoietic stem-cell transplantation (HSCT) and silicosis very rarely results in destroyed lung. Pneumoconiosis, which is highly prevalent in China, accounts for 9.2% of all lung transplants at our center, and silicosis has the highest incidence among all occupational diseases. In contrast, silicosis is a rare indication for LT in the United States, accounting for approximately 0.3% of all cases (13). The silicosis cohort was relatively young and exhibited better post-LT prognoses. Statistics from our center show that the 3-month, 1-year, and 3-year survival rates of patients with silicosis after lung transplantation were 90.6, 80.8, and 76.7%, respectively. BOS, alongside graft vs. host disease and pulmonary fibrosis, are common non-infectious pulmonary complications after HSCT. LT is currently the only effective treatment for patients who do not respond to drug therapy. According to the International Society for Heart and Lung Transplantation, the 3-month, 1-year, and 3-year cumulative survival rates of lung transplant patients with pulmonary complications after HSCT are 88, 79, and 64%, respectively (14). At present, there are no reports of patients with destroyed lung due to silicosis and BOS undergoing bilateral LT. In our study, we performed bilateral LT via clam-shell incisions in the supine position for the two patients with silicosis and BOS. Compared to patients with other diseases, the adhesions on the side of the destroyed lung were much lighter. ECMO was not used intraoperatively, and reduction of donor lung volumes was not required. The operations proceeded uneventfully, and both patients are still alive.

Destroyed lung results in thoracic deformity due to the consolidation and collapse of the lung on one side, and the resultant contralateral compensatory lung enlargement may lead to mediastinal displacement. Previous reports have attempted to use simultaneous contralateral pneumonectomy or lobectomy to reduce the size of the donor lung to fit into the recipient's thorax (15–17). For patients with severe intraoperative adhesions, surgery via an anterolateral incision in the lateral position can provide a better surgical field of view, making it easier to remove the destroyed lung. We recommended delaying chest closure on the side of the destroyed lung to maintain the patient's intraoperative oxygenation, especially in the absence of ECMO. Due to extensive adhesions, the surgical difficulty is significantly increased, which may result in a higher volume of blood loss and a longer operation time than surgeries performed in the supine position.

For patients without severe chest adhesions, we recommend performing bilateral LT via a clam-shell incision in the supine position without pneumonectomy or lobectomy. The mediastinum was restored to its original position by releasing the mediastinal adhesions, enabling the donor lung and the patient's chest to be size-matched. Consequently, lung lobe resection was avoided, and the lung's function was maximally preserved. Only one patient underwent size-reduced LT in the supine position group in this study, with the remaining two patients not requiring donor lung volume reduction via adhesion release. If there is a sufficient supply of donor lungs, bilateral LT is preferred over single LT, particularly among young patients with a longer life expectancy. Compared to single LT, bilateral LT has a longer survival period (18).

Due to the surgical difficulties inherent with destroyed lung, ECMO should be used instead of cardiopulmonary bypass in such patients to reduce surgical trauma and bleeding. Moreover, ECMO can mitigate the risk of postoperative primary graft dysfunction and tracheostomy compared to cardiopulmonary bypass, and both tracheal intubation time and postoperative in-hospital stay can be shortened (19). More importantly, ECMO can be continued postoperatively. For patients undergoing surgery in the lateral position, venovenous ECMO or V-A ECMO can be performed through the neck and femoral vessels. For patients undergoing surgery in the supine position, central ECMO cannulation can be performed through the right atrium and ascending aorta to maintain stable hemodynamics if there are unstable intraoperative levels of circulating blood oxygen. However, ECMO is not necessary for all patients with destroyed lung during surgery, only for those with severe pulmonary hypertension and intraoperative hemodynamic instability. Only one patient in our series required intraoperative ECMO; in other cases, the surgeries were completed successfully without the use of ECMO.

Our study has some limitations, including its small sample size and retrospective nature, that impact the generalizability of the findings. Hopefully, these findings will serve as a basis for future research, including long-term follow-up studies with improved serial quality. Despite this study's limitations, our experience can still be a valuable reference for thoracic surgeons to identify the best type of surgery for such patients.

## Conclusion

In summary, our experience indicates that bilateral LT is feasible for eligible patients with destroyed lung and asymmetric chest deformity. Adequate blood preparation and careful surgical dissection can markedly improve surgical success rates. By releasing the mediastinal adhesions and restoring the mediastinum through a clam-shell incision in the supine position, the need for pneumonectomy or lobectomy is not indicated, thus enabling maximal preservation of donor lung function. ECMO is not necessary for all patients, and the surgical method should be selected after careful consideration of the patient's condition.

## Data Availability Statement

The original contributions presented in the study are included in the article/supplementary material, further inquiries can be directed to the corresponding author/s.

## Ethics Statement

The studies involving human participants were reviewed and approved by The Institutional Ethics Committees of Wuxi People's Hospital. The patients/participants provided their written informed consent to participate in this study.

## Author Contributions

BY and JC designed and performed the study. JH, YJ, and DL collected and analyzed data. BY and JC wrote the paper. FL and SY supervised the clinical research and revised the manuscript. All authors approved the final manuscript.

## Conflict of Interest

The authors declare that the research was conducted in the absence of any commercial or financial relationships that could be construed as a potential conflict of interest.

## Publisher's Note

All claims expressed in this article are solely those of the authors and do not necessarily represent those of their affiliated organizations, or those of the publisher, the editors and the reviewers. Any product that may be evaluated in this article, or claim that may be made by its manufacturer, is not guaranteed or endorsed by the publisher.
